# Global Handwashing Day — October 15, 2014

**Published:** 2014-10-10

The 7th annual Global Handwashing Day will be observed October 15, 2014. This observance increases awareness and understanding of handwashing with soap as an effective and affordable way to prevent disease around the world.

Handwashing with soap has an important role to play in child survival and health. Approximately 2.2 million children aged <5 years die each year from diarrheal diseases and pneumonia, the top two causes of death among young children globally (1). Handwashing with soap can reduce the incidence of diarrhea among children aged <5 years by 30% (2) and the incidence of respiratory infections by 21% (3).

Although persons around the world clean their hands with water, few use soap to wash their hands. Washing hands with soap removes bacteria much more effectively (4).

Additional information on Global Handwashing Day is available from CDC at http://www.cdc.gov/features/globalhandwashing. General handwashing information is available from at http://www.cdc.gov/handwashing. Information on water-related hygiene is available at http://www.cdc.gov/healthywater/hygiene/index.html.
